# The early maternal environment shapes the parental response to offspring UV ornamentation

**DOI:** 10.1038/s41598-021-00251-4

**Published:** 2021-10-21

**Authors:** Jorge García-Campa, Wendt Müller, Ester Hernández-Correas, Judith Morales

**Affiliations:** 1 Department of Evolutionary Ecology, National Museum of Natural Sciences-Spanish National Research Council (CSIC), c/ José Gutiérrez Abascal 2, 28006 Madrid, Spain; 2 Department of Biology, Behavioural Ecology and Eco-Physiology Group, University of Antwerp, Universiteitsplein 1, Wilrijk, 2610 Antwerp, Belgium

**Keywords:** Ecology, Behavioural ecology, Evolutionary ecology

## Abstract

Parents allocate resources to offspring to increase their survival and to maximize their own fitness, while this investment implies costs to their condition and future reproduction. Parents are hence expected to optimally allocate their resources. They should invest equally in all their offspring under good conditions, but when parental capacity is limited, parents should invest in the offspring with the highest probability of survival. Such parental favouritism is facilitated by the fact that offspring have evolved condition-dependent traits to signal their quality to parents. In this study we explore whether the parental response to an offspring quality signal depends on the intrinsic capacity of the parents, here the female. We first manipulated the intrinsic capacity of blue tit (*Cyanistes caeruleus*) females through lutein-supplementation during egg laying, and we subsequently blocked the UV/yellow reflectance of breast feathers on half of the nestlings in each brood. We did not find evidence that the female intrinsic capacity shaped parental feeding or sibling competition according to offspring UV/yellow colouration. However, nestling UV/yellow colour affected costly behavioural interactions in the form of prey-testings (when a parent places a prey item into a nestling’s gape but removes it again). In lutein-supplemented nests, fathers but not mothers favoured UV-blocked chicks by testing them less often, supporting previous results. Accordingly, in lutein-supplemented nests, UV-blocked nestlings gained more mass than their siblings, while in control nests we found the opposite effect and UV-blocked nestlings gained less. Our results emphasize that the prenatal environment shaped the role of offspring UV/yellow colour during certain family interactions and are indicative for sex-specific parental care strategies.

## Introduction

Parents invest in their progeny in order to increase the offspring chances of survival, but resources are limited^1^. Hence, resource allocation to current offspring entails costs to parents in terms of reduced own survival and future reproductive prospects^2^. How parents optimally allocate their resources may not only vary across breeding events, but also among offspring of the same brood, at least in species raising more than one offspring at a time. When conditions are favourable (e.g., in benign environments and under high resource availability), the optimal strategy that guarantees the survival of all the offspring is that parents feed all the young equally^3,4^ or favour the offspring with the highest need^5^. However, under harsh conditions, individuals possess reduced parental capacities, which might not be enough to raise all the offspring. Then, parents are expected to bias their investment to offspring in better condition that will return greater fitness benefits^6^, thus following a brood reduction strategy^7,8^. Yet, this requires that parents assess offspring quality, which they could do based on the expression of signalling traits, such as behavioural (e.g., vocal or postural begging displays^9,10^) or structural traits (like the colouration of plumage, scales and skin^11,12^).

Signals of quality are frequent in many taxa and the expression of these traits can be associated with individual quality in several contexts, as occurs with sexual selection^13,14^. A couple of paradigmatic examples are the tail feathers of male peacocks or male deer antlers, and one main mechanism proposed to ensure the honesty of such signals is that they are costly to produce and maintain^15,16^. Interestingly, however, it is evident that signalling can play a significant role in other non-sexual contexts, including the period of parental care^17^. Here, both offspring and parents are potential bearers and receivers of signals of quality expressed by other family members^18,19^. For instance, one of the most common offspring signals is begging behaviour (i.e., solicitation of food from parents by means of postures and vocalizations), which dynamically transmits information about offspring state and need to both parents^20^ and siblings^21^. During these family interactions offspring may also display conspicuous structural traits such as colourful scales, gapes or feathers to trigger a parental response^22,23^. Honesty is again achieved because offspring pay a cost for expressing deceptive signals or for displaying or maintaining signalling traits that prevent cheating (honest signalling models^24^).

A well-known example of offspring signalling traits with a significant role in parent–offspring communication is the ultraviolet (UV) colouration of skin and other traits like beaks and feathers. Studies in various bird species showed that nestling UV skin colouration reliably reflects body mass and skeletal size (see^25^, in alpine swifts *Tachymarptis melba* and European starlings *Sturnus vulgaris*), as well as immune responsiveness (see^26^, in European starlings). Body mass is also correlated with nestling UV gape (see^23^ in barn swallows *Hirundo rustica*) and feather colouration (see^27,35^ in blue tits *Cyanistes caeruleus*). However, it has been argued that the parental response to offspring signalling traits may vary according to the current circumstances^28^. Indeed, evidence suggests that parents favour nestlings with enhanced UV colour as the breeding season progresses—once the resources become limiting^25^. However, it has been little explored experimentally whether parents favour specific offspring within a brood according to both the expression of offspring UV coloured signals and their own parental capacity, which is mainly constrained by resource availability. It has in addition to be considered that the expression of signalling traits may be shaped by multiple receivers^29^.

In this study, we investigate whether parental care preferences change with the expression of an offspring quality signal, and whether parental preferences for signal expression vary with the rearing capacity of the parents. To test our hypothesis, we first experimentally manipulated the availability of a specific micronutrient (i.e., lutein) for blue tit females at egg laying, which is the most energy-demanding stage of the females` life. Indeed, we have previously found that lutein supplementation facilitated egg laying, thus improving the female’s intrinsic capacity^30^. Additionally, we experimentally manipulated in half of the nestlings within each brood a nestling quality signal, the UV/yellow breast plumage colouration^35^. This trait mediates costly behavioural interactions among family-members, since blue tit nestlings with experimentally reduced UV reflectance beg more during parent–offspring and sib-sib competitive events and are in lower condition^29^. Also, when conditions are harsh, fathers but not mothers respond to offspring UV colour by performing more prey-testings, which occur when parents introduce a prey item in a nestling gape and then withdraw it again. This behaviour has been interpreted as a way to assess individual offspring need or hunger levels (see “Behavioural variables” section below) and as food is withdrawn, it imposes a cost to chicks in terms of reduced body mass gain^29^.

We expected that females with enhanced intrinsic capacity (i.e., lutein-supplemented females) should preferentially feed the offspring signalling poor quality (i.e., UV-blocked plumage colouration) to allow them to catch up in growth with their siblings (brood survival strategy). We also expected that lutein supplemented females would favour UV-blocked nestlings by reducing the number of prey testings. On the contrary, control females with a more limited capacity should mainly favour high-quality offspring (brood reduction strategy) by feeding them more and prey-test them less, which should be reflected in a significant body mass difference between UV-blocked and non-UV-blocked nestlings. As the males’ reproductive investment will likely depend on their partners intrinsic capacity, we expect that males co-adjust their behaviour to the females’ feeding strategies. Finally, we also expected that UV-blocking would play a role during sibling competition, in particular in the absence of the parents when begging is directed to and perceived only by siblings. Here, begging has been interpreted as a nestling strategy to negotiate future access to food when parents arrive at the nest again^31^. So we expected UV-blocked nestlings to beg more, as previously found in our study population^29^, and especially so in control nests, in which the females’ rearing capacity was more limited.

## Material and methods

### Ethics statement

All the methods were performed in accordance with the Spanish laws in relation to animal research. The study licenses to perform the experimental protocols were approved by the Spanish Research Council (CSIC, ref. 639/2017) and the Consejería de Medio Ambiente, Administración Local y Ordenación del Territorio, Comunidad de Madrid (ref. 10/056536.9/18; PROEX 237/17). The study was conducted in compliance with the ARRIVE guidelines.

### General methods and study species

This study was carried out in the locality of Miraflores de la Sierra (Madrid, Spain, 40°48′41.07″ N, 3°46′57.66″ O) during the spring of 2017. The study area embraces 187 nest-boxes spread out in a deciduous forest mainly dominated by Pyrenean oak (*Quercus pyrenaica*) at 1250 m of elevation. We studied a breeding population of blue tits, a territorial-monogamous passerine that in our study area only raises one clutch per season. Brood size is large (in our study population, on average 9.6 eggs ± 1.8 SD; n = 464 clutches; range 4–15). Both adults and offspring express colourful feather traits that are known to function as signals of quality. The most studied one is the UV reflectance of blue crown feathers in adults (e.g.^32,33^), which is not expressed in the offspring. However, the UV reflectance of yellow breast feathers is expressed both by parents^34^ and offspring^29^, and has been shown to reflect different aspects of individual quality^27,34,35^.

At the start of the breeding season, we visited nest-boxes every two days to record the beginning of nest construction, laying date and hatching date (day 0). Once nest construction was finished in a given nest, that is, when the moss cup was well defined but not filled with feathers and hair, we started lutein supplementation in that nest (see *Lutein supplementation* section below; for more details, see also^30^). We continued supplementing blue tit females during egg laying and finished once incubation started. Lutein is the main carotenoid pigment present in the birds’ plasma^36^ and eggs^37^, and is crucial for offspring development and feather colouration^38,39^.

Lutein-supplemented females completed their clutch faster than control females, as the treatment reduced the occurrence of egg-laying interruptions^30^. Two days before the expected hatching date, we performed a full-brood cross-fostering by exchanging clutches between nests in a fashion that allowed both lutein-supplemented females (n = 24) and control females (n = 23) to raise a control clutch. That is, the original clutches laid by lutein-supplemented females were raised in other nests not included in the present study. The rationale behind using only control clutches raised by both types of females was to exclude the influence of early maternal effects (i.e., differential carotenoid allocation into eggs) on offspring development.

In the second week after hatching (days 9–12), we trapped adults in their nest-boxes and marked the first one captured on the back feathers with a white marker (Edding 751; code 049), which allowed us to distinguish parents during video observations. On day 12, we ringed the nestlings, measured their body mass using a Pesola spring balance (to the nearest 0.01 g) and marked them individually on the head with the same white marker used for adults. We also collected 3–5 breast feathers per chick for molecular sexing (see Supplementary material). On day 12, we also substituted the original nest-box by a recording nest-box to familiarize parents with the set-up before the video recordings started. On day 13, we placed a night-vision video camera on the recording nest box (DX, 8 LED and 180° vision, China) and recorded the behaviour of all family members for 1.5 h. Just after video recording, we manipulated offspring UV colour within nests (see “Experimental manipulation of offspring UV feather reflectance in all nests” below). On day 14, we again recorded the behaviour for 1.5 h to assess the behavioural change of family members according to offspring UV colour and lutein-supplementation. At the end of the second video recording, we once more weighed all nestlings. We then calculated body mass change from days 12 to 14.

### Lutein supplementation prior and during egg laying

At the end of nest construction, we visited nests every two days and lutein was supplied in experimental nests using a dose of 50 mg of Versele Laga Yel-lux Oropharma (lutein 8000 mg/kg), which corresponds to 0.4 mg of lutein and which is within the natural limits consumed by blue tit females (for a detailed explanation, see^30^). Each dosage was mixed with 5 g of commercial bird fat with nuts (GRANA Oryx), whereas the same amount of bird fat without lutein was provided to nests in the control treatment. We confirmed through direct observations that males rarely visited the nest during nest construction and we can thus assume that the supplement was mainly consumed by females^30^.

### Experimental manipulation of offspring UV feather reflectance in all nests

Prior to UV colour manipulation, we measured the original UV reflectance of nestling yellow breast feathers with a portable spectrophotometer (Jazz, OceanOptics©). UV Chroma was calculated as the reflectance in the UV wave-band region of the spectrum divided by the total reflectance of the spectrum in the avian visual range (R_300–400_/R_300–700_), following Johnsen et al.^27^. Original nestling UV/yellow chroma did not differ between control and lutein-supplemented nests (F_1,42.8_ = 0.00; *P* = 0.95) nor between UV-blocked and non-UV-blocked siblings within nests (F_1,300_ = 1.29; *P* = 0.26).

On day 13, we recorded behaviour for the first time and, as soon as the video-recording was finished, we experimentally reduced the UV reflectance of yellow breast feathers in half of the nestlings in each nest using a yellow marker (Edding 4500; code 005). We randomly assigned the non-UV-blocked and UV-blocked treatment to the first nestling to be handled in each nest and then we alternated treatments when processing the rest of the brood. This manipulation has been successfully applied previously to both blue tit nestlings (^29^; see^40^, in great tits, *Parus major*) and adults^34^. By reducing the UV/yellow reflectance, half of the nestlings in each nest resembled individuals in poor body condition. We applied the same marker to non-UV-blocked siblings but in the lower part of the wing feathers. This was done to control for potential undesired side-effects of the marker but in a similar-sized region that cannot be seen by other family members. Nonetheless, in previous studies, we have not detected side-effects of the markers on blue tit health or behaviour^29^.

### Behavioural variables

We recorded the behaviour of all family members individually during 30 min (excluding the first half an hour and the last 10 min of the video to avoid possible interferences due to placing or removing the camera). Feeding rates recorded during 30 min are highly correlated with those recorded during 1 h (Pearson's r = 0.84, *P* < 0.001, n = 45 nests, data from^29^). We recorded parental decisions in the form of feeding rates and prey-testings. The latter occur when parents introduce a prey item in a nestling gape and then remove it again^41,42^, and it has been proposed as a strategy used by parents to assess individual offspring hunger levels, since it triggers begging^29^. Prey testings—or prey withdrawal—are thus like a “hunger test” that might be especially important under conditions of low food availability. Moreover, in the study population, prey-testings have been found to impose a cost to nestlings in terms of growth^29^.

We also recorded sibling competition, which was measured as parent-absent begging, when begging can only be perceived by siblings (see for instance^21^). Each time the parents had left the nest, we waited 30 s and then recorded parent-absent begging during another 30 s. For scoring begging behaviour, we followed a 4-point scale adapted from Kölliker et al.^43^: 0 = calm, 1 = weak gapping, 2 = gapping and neck stretched, 3 = gapping, neck stretched and standing, 4 = gapping, neck stretched, standing and wing flapping (see also^29^). We obtained the behavioural data of individual nestlings (i.e., number of prey items and prey-testings received, as well as the begging levels during parent-absent events). Then, for each behavioural variable we calculated the change between final (post-UV treatment) and initial values (pre-UV treatment). Observers always were unaware of female treatment and nestling UV treatment. Moreover, the sex of the parents was unknown for the observers, who only distinguished between presence/absence of an adult’s back mark.

We were able to record both pre- and post-UV treatment behaviour in 32 nests. Sample sizes differ when the behaviour of fathers and mothers was analysed separately, since the father appeared in both videos in 24 nests and the mother in 29 nests. Finally, we lacked visibility to score prey testings and nestling begging intensity in certain nests when focal nestlings were not visible to the observers.

### Statistical analyses

We used SAS 9.4. (SAS Inst., Cary, NC, USA) for all statistical analyses. Since we were interested in the interaction term between both treatments to address the main study question (how parental care strategies depend on UV/yellow of nestlings and how these vary with the availability of lutein for blue tit laying females), models were not simplified and we thus present full models including the interaction. Models were checked for residual normality using a Shapiro Wilk tests. Furthermore, all tests were conducted using a Type III sum of squares. All mixed models included a random intercept (nest ID) and a random slope (nest ID × nestling UV treatment) in order to account for the fact that half of the nestlings in each nest were UV-blocked and the other half were not.

We analysed the number of prey-testings and the number of prey items received by each nestling using generalized mixed models (GLIMMIX procedure in SAS with Poisson error structure). In these models we included female treatment, nestling UV treatment, nestling sex, brood size, hatching date and the interaction between both treatments as fixed effects. We ran a linear mixed model (MIXED procedure in SAS) to analyse parent-absent begging with normal error. In this model, we included the same variables as above.

Finally, we examined whether body mass change (log_10_ transformed) was affected by the treatments with a linear mixed model that included the same variables as above.

## Results

**Figure 1:**
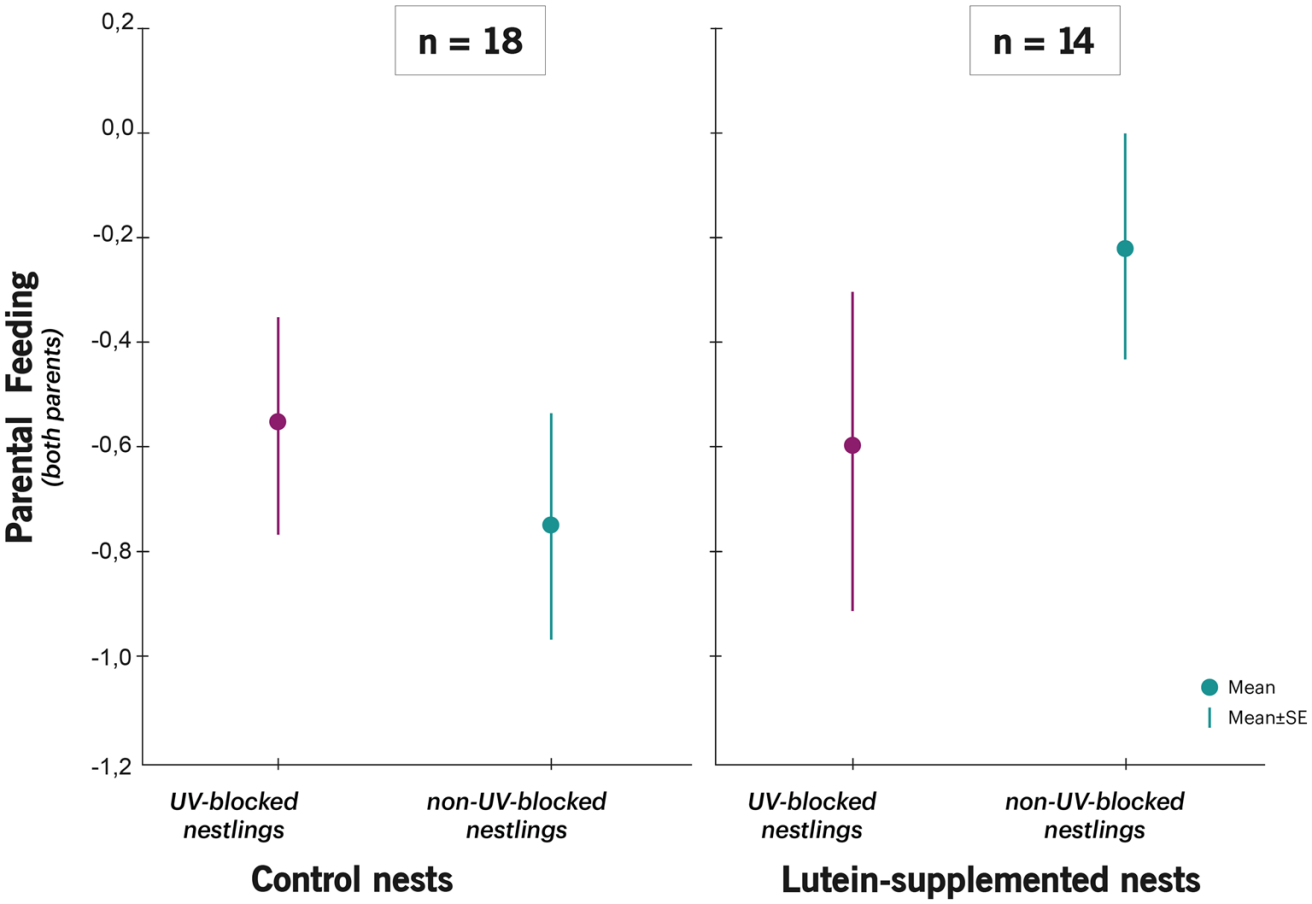
Difference in parental feeding rates (Post UV manipulation − Prior UV manipulation) according to nestling UV colour manipulation and female supplementation treatment. Values are (mean ± SE) residuals plus the average difference in feeding rates from a model that includes all variables except the interaction between both treatments. Sample sizes for control females and lutein-supplemented females are shown.

**Figure 2:**
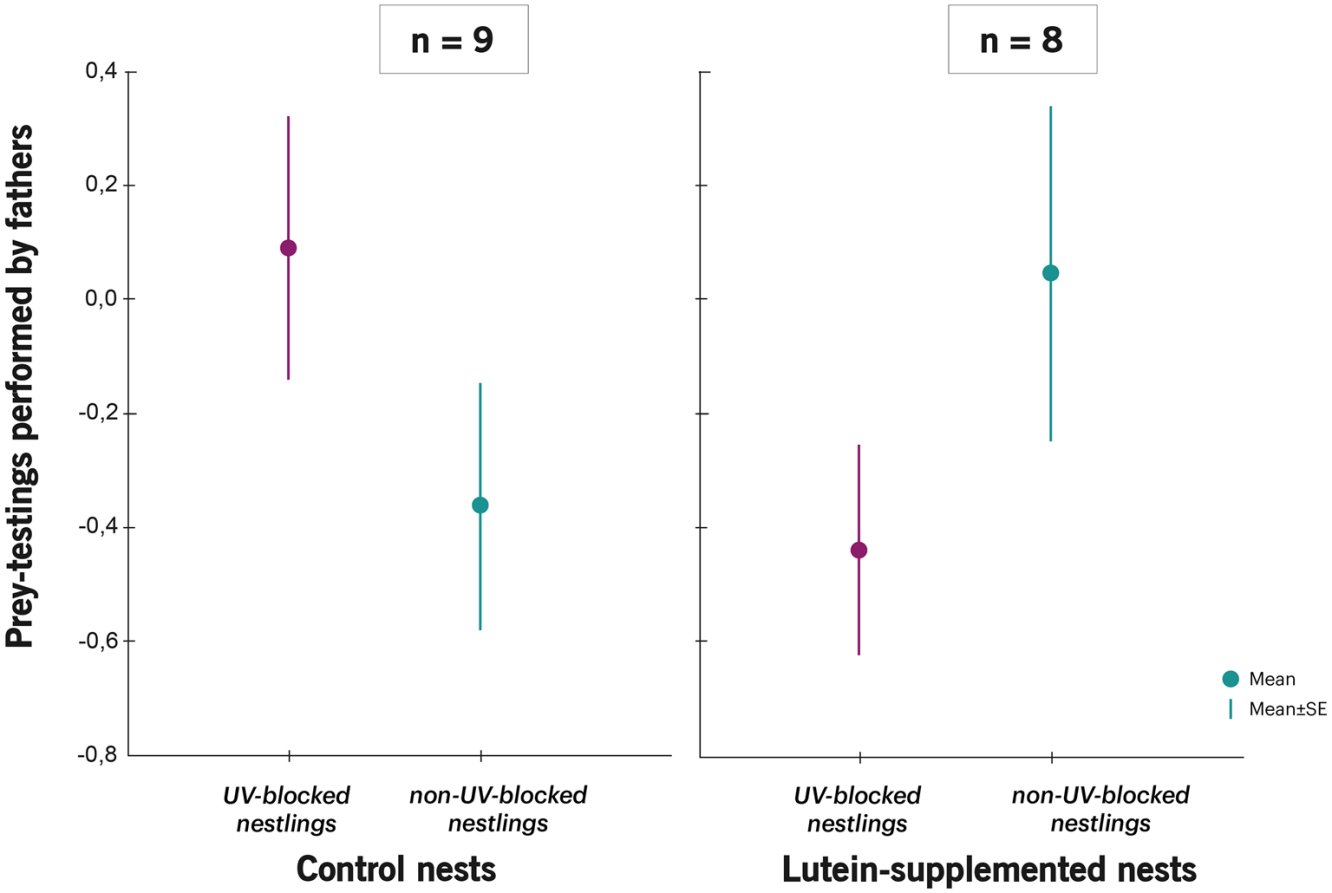
Difference (mean ± SE) in prey-testings performed by males (Post UV manipulation − Prior UV manipulation) according to nestling UV colour manipulation and female supplementation treatment. Values are (mean ± SE) residuals plus the average difference in male prey-testings from a model that includes all variables except the interaction between both treatments. Sample sizes for control females and lutein-supplemented females are shown.

Feeding rates of males and females were not affected by the interaction between both treatments (Table 1; Fig. 1). However, we found that the number of prey-testings performed by males, but not by females, were indeed affected by the interaction between lutein and UV treatment (Table 2). In lutein-supplemented nests, males prey-tested UV-blocked chicks less often than their non-UV-blocked siblings (coef. = − 1.24 ± 0.57, F_1,57_ = 4.97; *P* = 0.033), while in control nests, males did not prey-test chicks differently according to UV treatment (coef. = − 0.88 ± 0.53, F_1,57_ = 4.97; *P* = 0.11 Fig. 2). This interaction effect remained significant if begging intensity in parent-absent was controlled for in the model (F_1,56_ = 5.41; *P* = 0.024).

**Table 1:** Mixed models showing the effects of nestling UV treatment (non-UV-blocked/UV-blocked feather colouration) and female treatment at laying (control/lutein-supplemented) on the number of preys provided by both parents, and by males and by females separately. Coefficients are shown for control nests, non-UV-blocked nestlings and females.

	Parental feeding	Female feeding	Male feeding
Intercept	*Coef* = − 0.61 ± 1.68	*Coef* = 1.70 ± 2.28	*Coef* = − 2.92 ± 2.87
Female treatment (control)	*Coef* = − 0.24 ± 0.28 *F*_1,66_ = 1.83 *P* = 0.18	*Coef* = − 0.23 ± 0.41 *F*_1,67_ = 0.69 *P* = 0.41	*Coef* = − 0.05 ± 0.37 *F*_1,55_ = 0.26 *P* = 0.61
Nestling UV treatment (non-UV-Blocked)	*Coef* = − 0.06 ± 0.25 *F*_1,25_ = 0.36 *P* = 0.55	*Coef* = 0.12 ± 0.36 *F*_1,24_ = 0.13 *P* = 0.72	*Coef* = 0.05 ± 0.31 *F*_1,20_ = 0.07 *P* = 0.80
Nestling sex (females)	*Coef* = 0.02 ± 0.18 *F*_1,66_ = 0.01 *P* = 0.92	*Coef* = 0.21 ± 0.23 *F*_1,67_ = 0.79 *P* = 0.38	*Coef* = − 0.02 ± 0.22 *F*_1,55_ = 0.01 *P* = 0.92
Hatching date	*Coef* = − 0.003 ± 0.04 *F*_1,66_ = 0.01 *P* = 0.94	*Coef* = − 0.06 ± 0.05 *F*_1,67_ = 1.03 *P* = 0.31	*Coef* = 0.03 ± 0.06 *F*_1,55_ = 0.26 *P* = 0.61
Brood size	*Coef* = 0.11 ± 0.08 *F*_1,66_ = 1.71 *P* = 0.20	*Coef* = − 0.03 ± 0.11 *F*_1,67_ = 0.09 *P* = 0.77	*Coef* = − 0.23 ± 0.15 *F*_1,55_ = 2.29 *P* = 0.14
Female treat. × Nestling UV treat	*Coef* = − 0.11 ± 0.36 *F*_1,66_ = 0.09 *P* = 0.77	*Coef* = − 0.07 ± 0.46 *F*_1,67_ = 0.02 *P* = 0.88	*Coef* = − 0.22 ± 0.43 *F*_1,55_ = 0.26 *P* = 0.61

**Table 2:** Mixed models showing the effects of nestling UV treatment (non-UV-blocked/UV-blocked feather colouration) and female treatment at laying (control/lutein-supplemented) on the number of prey-testings performed by parents, the parent-absent begging intensity and the (log) body mass change. Coefficients are shown for control nests, non-UV-blocked nestlings and females. Significant differences are marked in bold. In Supplementary material, we show the mixed model of body mass change (not log transformed).

	Prey-testings performed by females	Prey-testings performed by males	Parent-absent begging	Body mass change
Intercept	*Coef* = − 6.57 ± 6.50	*Coef* = 0.08 ± 7.09	*Coef* = 0.62 ± 1.05	*Coef* = 0.35 ± 0.21
Female treatment (control)	*Coef* = 0.12 ± 0.94 *F*_1,70_ = 0.00 *P* = 0.99	*Coef* = 1.03 ± 0.78 *F*_1,57_ = 0.15 *P* = 0.70	*Coef* = 0.003 ± 0.12 *F*_1,17.9_ = 0.08 *P* = 0.77	*Coef* = -0.02 ± 0.03 *F*_1,42.6_ = 0.32 *P* = 0.57
Nestling UV treatment (non-UV-Blocked)	*Coef* = 0.61 ± 0.68 *F*_1,18_ = 1.15 *P* = 0.30	*Coef* = 0.83 ± 0.49 *F*_1,14_ = 0.06 *P* = 0.81	*Coef* = − 0.004 ± 0.099 *F*_1,127_ = 0.11 *P* = 0.74	*Coef* = -0.05 ± 0.02 *F*_1,293_ = 1.51 *P* = 0.22
Nestling sex (females)	*Coef* = − 0.54 ± 0.27 *F*_1,70_ = 4.04 *P* = 0.05	*Coef* = − 0.25 ± 0.27 *F*_1,57_ = 0.86 *P* = 0.36	*Coef* = − 0.10 ± 0.07 *F*_1,138_ = 2.31 *P* = 0.13	*Coef* = 0.01 ± 0.01 *F*_1,305_ = 0.19 *P* = 0.66
Hatching date	*Coef* = 0.19 ± 0.16 *F*_1,70_ = 1.30 *P* = 0.26	*Coef* = 0.01 ± 0.16 *F*_1,57_ = 0.01 *P* = 0.94	*Coef* = 0.003 ± 0.025 *F*_1,19.6_ = 0.02 *P* = 0.90	*Coef* = − 0.003 ± 0.005 *F*_1,42.7_ = 0.23 *P* = 0.64
Brood size	*Coef* = − 0.15 ± 0.24 *F*_1,70_ = 0.40 *P* = 0.53	*Coef* = − 0.21 ± 0.26 *F*_1,57_ = 0.64 *P* = 0.43	*Coef* = − 0.09 ± 0.04 *F*_1,24.1_ = 6.58 ***P* = 0.017**	*Coef* = 0.003 ± 0.010 *F*_1,46.4_ = 0.09 *P* = 0.76
Female treat. × Nestling UV treat	*Coef* = − 0.20 ± 0.94 *F*_1,70_ = 0.05 *P* = 0.83	*Coef* = − 1.51 ± 0.68 *F*_1,57_ = 4.97 ***P* = 0.029**	*Coef* = − 0.05 ± 0.13 *F*_1,127_ = 0.15 *P* = 0.70	*Coef* = -0.07 ± 0.03 *F*_1,293_ = 6.81 ***P* = 0.0095**

We did not find differences in parent-absent begging, thus in sibling negotiation/competition, according to the interaction between both treatments (Table 2).

**Figure 3:**
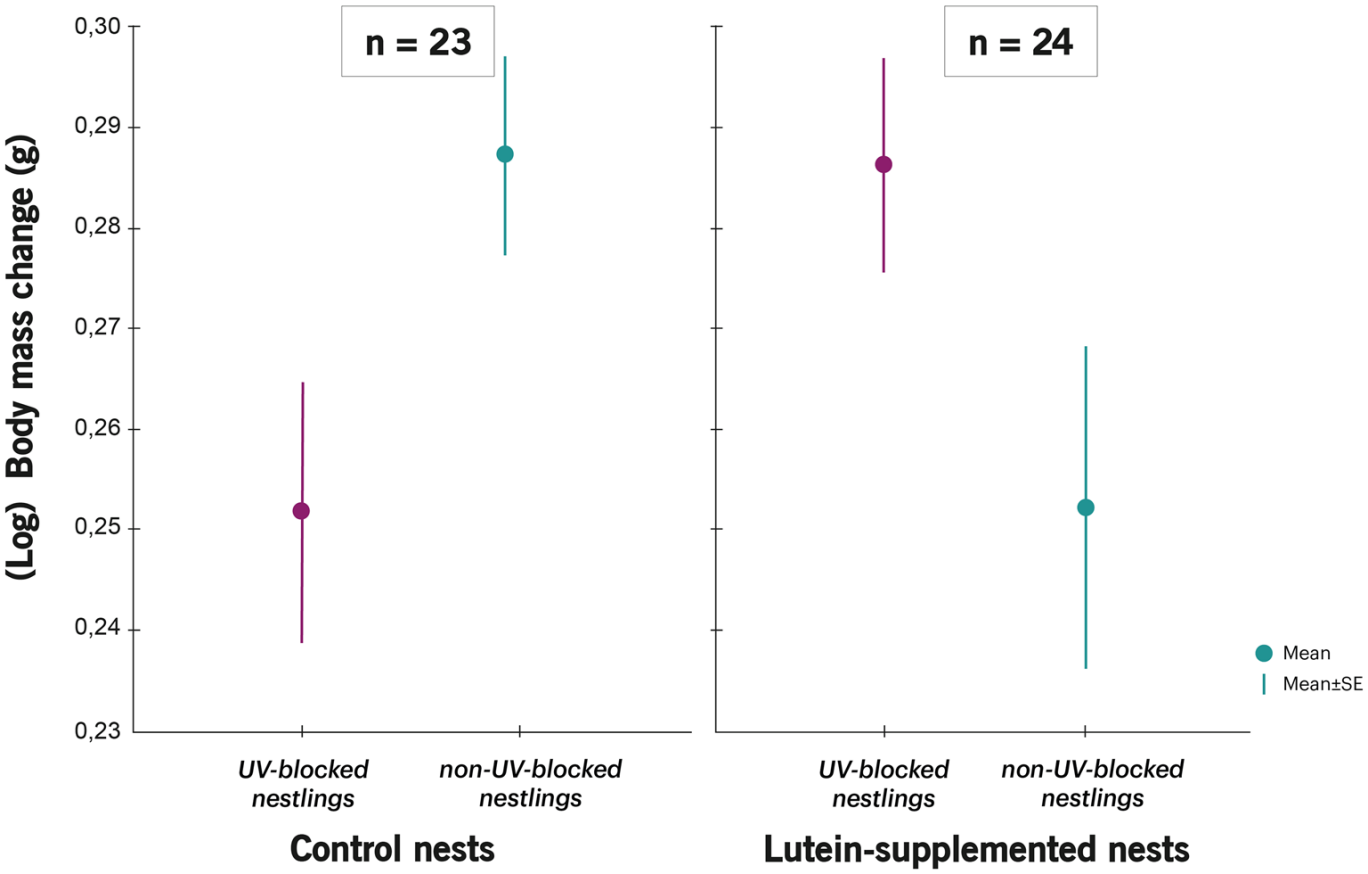
Nestling (log_10_) body mass change (mean ± SE) according to nestling UV manipulation and female supplementation treatment. Values are (mean ± SE) residuals from a model plus the average difference in (log_10_) body mass change that includes all variables except the interaction between both treatments. Sample sizes for control females and lutein-supplemented females are shown. See in Supplementary material the figure for body mass change (not log transformed).

However, as expected, there was a significant effect of the interaction between both treatments on nestling body mass change (Table 2). In lutein-supplemented nests, UV-blocked nestlings gained more body mass than their non-UV-blocked siblings (coef. = 0.29 ± 0.023, F_1,293_ = 6.81; *P* < 0.001), while in control nests, they gained less (coef. = 0.29 ± 0.023, F_1,293_ = 6.81; *P* < 0.001) (Fig. 3; see also Figure S1).

## Discussion

Our results suggest that the UV/yellow colouration of nestlings does not affect parental feeding or sibling competition (i.e., parent-absent begging) according to the availability of lutein during egg laying. However, males of lutein-supplemented nests favoured UV-blocked nestlings by testing them less often than their non-UV-blocked siblings. This was partly mirrored in nestling body mass change. UV-blocked nestlings gained more body mass than their siblings but only in lutein-supplemented nests, while in control nests UV-blocked nestlings gained less. Therefore, nestling UV/yellow colour modulated certain intra-family interactions according to the quality of the prenatal environment (and thus to the females’ intrinsic capacity^30^), which ultimately affected offspring growth.

There were no significant differences in parental feeding behaviour between UV-blocked and non-UV-blocked nestlings, independently of lutein availability in the prenatal environment. Thus, neither males nor females preferentially fed high quality offspring (i.e., non-UV-blocked nestlings), while we expected such parental favouritism to arise when rearing capacity was insufficient to raise all the brood^4^ (here, control broods in which females did not receive the lutein supplementation prior to laying). Although supplemented females laid their clutch faster^30^, we did not detect an effect on female body mass at the end of the nestling period (F_1,70_ = 0.10; *P* = 0.76), around one month after supplementation (note that capturing females at early stages increases the risk of nest desertion). Thus we cannot prove that there was a difference in body mass between lutein-supplemented and control females at the moment of this experiment, which should be taken into account when interpreting the results. However, it is possible that the supplemented females were in better condition during egg laying but the effect vanished by the end of the nestling period, while there still might have been carry-over effects. Perhaps a handicapping manipulation such as feather clipping or a more drastic food deprivation experiment (e.g., by temporarily closing the nest-box entrance)^44,45^ would have resulted in significant differences in parental care strategies. Besides, we only observed thirty minutes of behaviour, which is a snapshot of intra-family interactions during the two days elapsed since UV manipulation took place.

Yet, half an hour of observation was enough to detect significant differences in the prey-testings performed by male parents. In lutein-supplemented nests, males tested UV-blocked nestlings (signalling poor condition) less often than their non-UV-blocked siblings, whereas there were no significant differences in control nests. Recently, prey-testings in blue tits have been interpreted as a parental strategy to evaluate nestling hunger levels^29^. Similar costly “hunger tests” have been found in other avian, mammal and insect species^46–49^ raising more than one offspring at a time and as result of parent–offspring conflict over parental care. Such tests are costly for the offspring since they commonly trigger offspring begging, usually through the expression of signals of parental quality (i.e., the bill red spot in some gull species^49,50^) or active behaviours (i.e., feeding races in penguins^47,51^). Hence, parents can evaluate the offspring true motivation of being fed and be more efficient in optimizing their investment (e.g., by shifting their care to the neediest sibling when rearing capacity is high). Forced energy expenditure could explain why more prey-testings impose a growth cost to blue tit nestlings^29^. A non-exclusive possibility is that prey-testings occur when nestlings have gapes not large enough to swallow big preys^41^. However, this interpretation cannot explain our results, since the occurrence of prey-testings and prey size were not correlated, neither prior to UV-blocking (r_32_ = − 0.21; *P* = 0.25) nor after it (r_20_ = 0.16; *P* = 0.53). Our results rather suggest that, under conditions of high resource availability, at least fathers were more inclined to favour UV-blocked nestlings without testing them. Interestingly, previous results in the same study population suggested that only males but not females modified prey-testings according to both nestling UV colour and food availability at the end of the nestling period^29^. Our results together with previous evidence point to the possibility that males are more responsive to nestling UV colouration than females. At current we can only speculate about the male-specific effects. During egg laying and incubation, males guard females and feed them frequently^52^, sometimes even up to 74 times per day^53^. So that an extended egg laying period would have been more costly for males. Still males and females may also apply different provisioning strategies^45,54^.

We also hypothesized that UV-blocked nestlings—those signalling poor condition—should beg more than their non-UV-blocked siblings when parents are absent, since they should try to discourage their siblings from competing for the next parental feeding (“sibling negotiation hypothesis”)^31^. However, we did not detect significant effects on sibling competition, in contrast to a previous study^31^. It is possible that environmental conditions were too favourable in the postnatal environment (well after lutein supplementation) so that differences on nestling need among UV treatments diminished.

Interestingly, however, in lutein-supplemented nests, UV-blocked nestlings gained more body mass than their non-UV-blocked siblings, but in control nests, the result was the opposite and UV-blocked nestlings gained less. Since all the offspring included in the experiment belonged to a clutch laid by a control female, we are certain that body mass change was not affected by early maternal effects but rather by behavioural interactions among family members. One likely possibility is that the father’s prey-testings mediated body mass change, and thus low-quality chicks gained more body mass because they were prey-tested less often in lutein-supplemented nests (see the contrasting patterns in Figs. 2 and 3). Besides, in control nests, UV-blocked offspring gained less body mass and tended (not significantly) to receive more prey-testings than their non-UV-blocked siblings. Thus, by reducing the prey-testings to UV-blocked nestlings in lutein-supplemented nests, fathers would be compensating for their low quality and facilitating a brood survival strategy.

In conclusion, our results suggest that offspring feather UV-colour mediates intra-family interactions and that this effect depends on the quality of the prenatal environment. Our findings thus support that offspring UV-colour functions as a signal, at least to male parents, which shaped their response to nestling UV colouration according to lutein availability during egg laying. This is in line with previous findings that sex-specific care strategies can vary with condition-dependent traits of the offspring. However, the effects we observed are comparatively small, while multiple traits have been studied and tested, and it may hence require more studies to provide further support to our findings.

## Supplementary Information

Supplementary Information.

## Data Availability

The datasets generated and analysed during the current study are available in: García-Campa, Jorge; Müller, Wendt; Ester, Hernández-Correas; Morales, Judith (2021): The early maternal environment shapes the parental response to offspring UV ornamentation. figshare. Dataset. 10.6084/m9.figshare.14054756.v1.
