# Supplementary material for: The early maternal environment shapes the parental response to offspring UV ornamentation
Source: Sci Rep. 2021 Oct 21;11:20808. doi: 10.1038/s41598-021-00251-4 (PMC8531375; doi:10.1038/s41598-021-00251-4)
Supplement: Supplementary file 1 — Supplementary Information. [file 41598_2021_251_MOESM1_ESM.docx]

**The early maternal environment shapes the parental response to offspring UV ornamentation**

**Supplementary material**

Jorge García-Campa^a*^, Wendt Müller^b^, Ester Hernández-Correas^a^ & Judith Morales^a^

^a^ Department of Evolutionary Ecology, National Museum of Natural Sciences – Spanish National Research Council (CSIC). c/ José Gutiérrez Abascal 2, 28006 Madrid, Spain. ^b^ Department of Biology, Behavioural Ecology and Ecophysiology Group, University of Antwerp, Universiteitsplein 1, 2610 Antwerp, Wilrijk, Belgium *Correspondence: jgarciacampa@gmail.com

**Molecular sexing of nestlings**

DNA was extracted from 25 mg of feather sheaths using the Qiagen DNeasy Blood and Tissue kit (Qiagen Inc, Valencia, CA, USA). Sex identification was performed by polymerase chain reaction (PCR) amplification of the CHD-W and CHD-Z genes with primers P2 and P8, following Griffiths et al. (1998) with a few modifications. An initial denaturizing step at 94°C for 4 min 30 s was followed by 40 cycles of 94°C during 30 s, 49°C during 45 s and 72°C during 45 s. A final run of 72°C during 10 min completed the program. Amplification was carried out in a total volume of 10 µl. Each PCR sample contained: 2 µl DNA, 0.08 µl *Taq* polymerase (TaKaRa BIO Inc, Japan), 0.8 µl dNTP 2.5 mM, 0.5 µl of each primer 10 µM, 1 µl of 10X PCR buffer and 5 µl of sterilized distilled water. The sex of 19 chicks from 14 nests could not be determined due to unsuccessful DNA extraction.

Griffiths R, Double MC, Orr K, Dawson JG. 1998. A DNA test to sex most birds. *Molecular Ecology* 7:1071-1075.

**Colour meassurements**

Colour was extracted after being measured on nestling yellow breast feathers with a portable spectrophotometer (Jazz, OceanOptics^©^) using CLR program v 1.1 (Montgomerie 2009).

Montgomerie R. 2009. CLR, version 1.1. Queen’s University, Kingston, Canada.

**Figure S1:** Nestling body mass (not log transformed) change (mean ± SE) according to nestling UV manipulation and female supplementation treatment. Values are (mean ± SE) residuals from a model that includes all variables except the interaction between both treatments plus the average difference in body mass change.


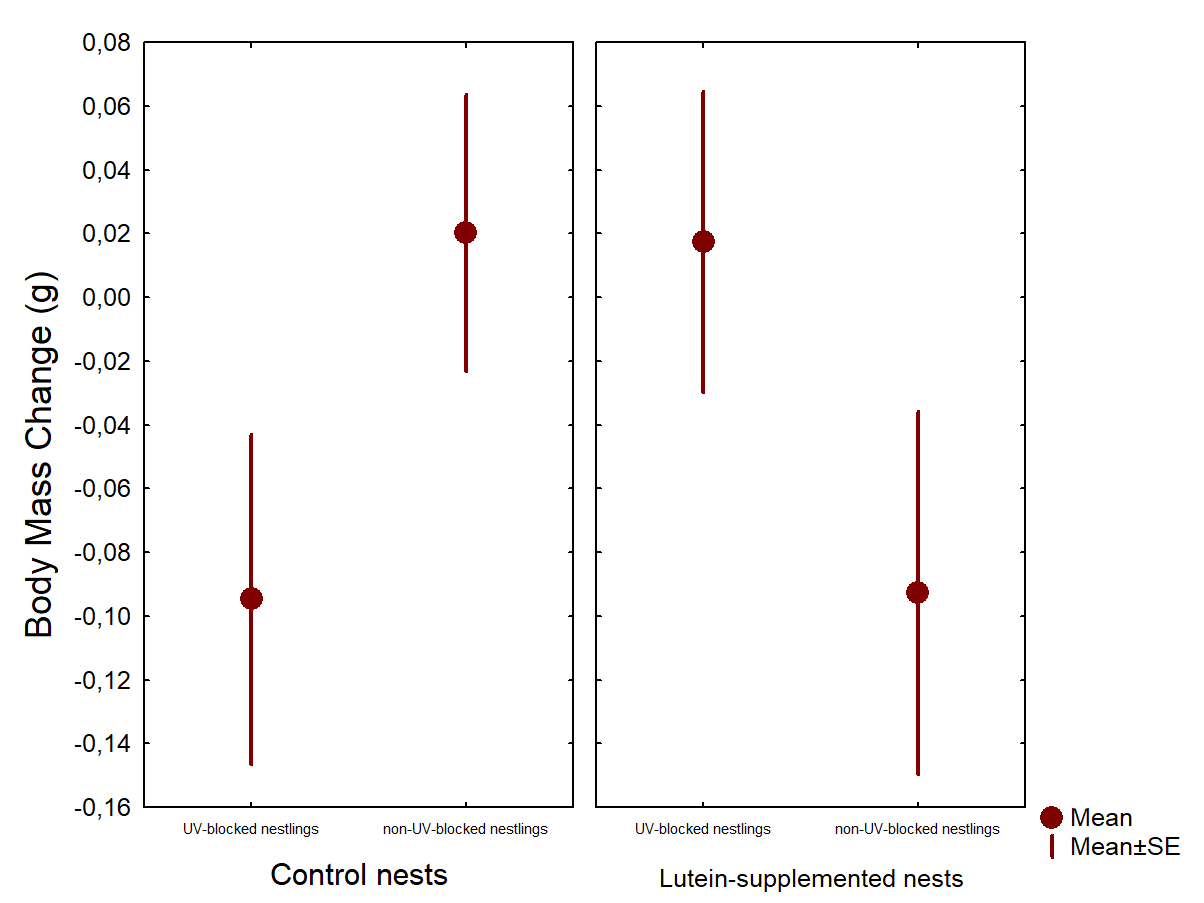


**S.1 Supplementary table:** Mixed models showing the effects of nestling UV treatment (non-UV-blocked/UV-blocked feather colouration) and female treatment at laying (control/lutein-supplemented) on the body mass change. Coefficients are shown for control nests, non-UV-blocked nestlings and females. Significant differences are marked in bold.

|  | ***Body mass change*** | |
| --- | --- | --- |
| ***Intercept*** | *coef* = 0.28 ± 0.91 |  |
| ***Female treatment***  *(Control)* | *coef* = -0.06 ± 0.14  *F*_1,42.4_ = 0.20  *P* = 0.66 | |
| ***Nestling UV treatment***  *(Non-UV-Blocked)* | *coef* = -0.06 ± 0.14  *F*_1,292_ = 0.51  *P* = 0.48 | |
| ***Nestling sex***  *(Females)* | *coef* = -0.15 ± 0.08  *F*_1,303_ = 0.01  *P* = 0.93 | |
| ***Hatching date*** | *coef* = -0.01 ± 0.02  *F*_1,42.5_ = 0.21  *P* = 0.65 | |
| ***Brood size*** | *coef* = 0.01 ± 0.04  *F*_1,45.7_ = 0.08  *P* = 0.79 | |
| ***Female treat. * Nestling UV treat.*** | *coef* = 0.23 ± 0.11  *F*_1,292_ = 4.48  ***P* = 0.035** | |
